# Pharmacokinetic modeling of gentamicin in treatment of infective endocarditis: Model development and validation of existing models

**DOI:** 10.1371/journal.pone.0177324

**Published:** 2017-05-05

**Authors:** Anna Gomes, Lars van der Wijk, Johannes H. Proost, Bhanu Sinha, Daan J. Touw

**Affiliations:** 1 Department of Medical Microbiology, University of Groningen, University Medical Center Groningen, Groningen, The Netherlands; 2 Department of Clinical Pharmacy and Pharmacology, University of Groningen, University Medical Center Groningen, Groningen, The Netherlands; 3 Department of Pharmacokinetics, Toxicology and Targeting, University of Groningen, Groningen, The Netherlands; University of Kentucky, UNITED STATES

## Abstract

Gentamicin shows large variations in half-life and volume of distribution (Vd) within and between individuals. Thus, monitoring and accurately predicting serum levels are required to optimize effectiveness and minimize toxicity. Currently, two population pharmacokinetic models are applied for predicting gentamicin doses in adults. For endocarditis patients the optimal model is unknown. We aimed at: 1) creating an optimal model for endocarditis patients; and 2) assessing whether the endocarditis and existing models can accurately predict serum levels. We performed a retrospective observational two-cohort study: one cohort to parameterize the endocarditis model by iterative two-stage Bayesian analysis, and a second cohort to validate and compare all three models. The Akaike Information Criterion and the weighted sum of squares of the residuals divided by the degrees of freedom were used to select the endocarditis model. Median Prediction Error (MDPE) and Median Absolute Prediction Error (MDAPE) were used to test all models with the validation dataset. We built the endocarditis model based on data from the modeling cohort (65 patients) with a fixed 0.277 L/h/70kg metabolic clearance, 0.698 (±0.358) renal clearance as fraction of creatinine clearance, and Vd 0.312 (±0.076) L/kg corrected lean body mass. External validation with data from 14 validation cohort patients showed a similar predictive power of the endocarditis model (MDPE -1.77%, MDAPE 4.68%) as compared to the intensive-care (MDPE -1.33%, MDAPE 4.37%) and standard (MDPE -0.90%, MDAPE 4.82%) models. All models acceptably predicted pharmacokinetic parameters for gentamicin in endocarditis patients. However, these patients appear to have an increased Vd, similar to intensive care patients. Vd mainly determines the height of peak serum levels, which in turn correlate with bactericidal activity. In order to maintain simplicity, we advise to use the existing intensive-care model in clinical practice to avoid potential underdosing of gentamicin in endocarditis patients.

## Introduction

Infective endocarditis is an infection of the endothelial lining of the heart, most commonly involving the valvular leaflets [1–5]. Infection can also involve intracardiac prosthetic material. The most common causative pathogens are staphylococci (42.1%), streptococci (29.6%), and enterococci (10.6%), but many microorganisms have been implicated in endocarditis [6–8]. Guidelines recommend the use of gentamicin combined with beta-lactams as antimicrobial treatment, mainly for Gram-positive pathogens [9,10]. Beta-lactams are thought to increase access of gentamicin to the bacterial cell membrane of Gram-positive micro-organisms, causing a synergistic effect [11].

Gentamicin, an aminoglycoside, is classified as bactericidal antimicrobial agent [6]. Its clinical efficacy is predicted by the pharmacodynamic factor C_max_:MIC, which is the ratio of the gentamicin peak level in serum (C_max_) and the minimum inhibitory concentration (MIC) of the micro-organism [12]. For systemic infections, gentamicin needs to be administered intravenously, displaying a short half-life of 2–3 hours [11–13]. This hydrophilic molecule has a volume of distribution (Vd) similar to the extracellular fluid, and a clearance proportional to the glomerular filtration rate (90% renal clearance) [12,14]. A small proportion of gentamicin is cleared non-renally (CLm; 10%).

Gentamicin peak and trough levels in endocarditis are aimed at 9–12 mg/l and <0.5–1 mg/l [15,16]. Therefore, body weight-based dosing is started with 3 mg/kg once daily [17,18]. Peak levels determine effectiveness of gentamicin treatment [19], with optimum bactericidal activity and prevention of resistance achieved with C_max_:MIC ≥10 [13]. Trough levels determine both ototoxicity (hearing loss, tinnitus) and nephrotoxicity (usually reversible), with decreasing renal clearance causing drug accumulation [12,19]. Toxicity also depends on patient characteristics (age, hydration status, blood pressure) and simultaneous medication with similar toxic potential [11,12,20], and toxicity earlier in the course of treatment is observed with higher peak levels (opposing previous reports [11,13]). Peak levels indeed correlate indirectly with nephrotoxicity, via the area under the serum concentration-time curve (AUC) [21,22].

A starting dose of 3 mg/kg gentamicin causes individual peak levels to vary, caused by large intra and inter individual variability of pharmacokinetic parameters (e.g. Vd). Therefore, therapeutic drug monitoring (TDM) is advised for optimal dosing, applying serum gentamicin levels as well as pharmacokinetic (PK) and pharmacodynamic principles [12,19]. Guiding dose and dosing interval maximizes efficacy and minimizes toxicity. TDM has been shown to decrease hospital stay, and the incidence of nephrotoxicity, mortality, costs [19]. Effects of TDM on ototoxicity are less clear [12].

To support TDM, population pharmacokinetic (PPK) models for gentamicin have been created using Bayesian statistics. These PPK models were created for different populations in order to enable appropriate advise on the dose of gentamicin to start with [23]. Thereafter, Bayesian feedback algorithms, using the model and associated errors, enable advise on dosage adjustments based on few measured serum levels [12,14]. As a number of factors influence the predicting ability of PPK models, standardizing protocols and region-specific use are recommended [19,23]. Importantly, patients admitted on the intensive care unit (ICU) are shown to have higher Vd (more extravasation of drugs), as compared with patients admitted to a standard ward. Therefore, two PPK models for dosing of gentamicin in adult patients are used in the Netherlands [24]: one with a high Vd (0.336 L/kg; ICU model) and the other with a lower Vd (0.273 L/kg; standard model), respectively.

Although the standard and ICU PPK models for gentamicin perform well [24], they have never been validated in endocarditis patients. Current dosing according to the standard model often results in insufficient peak levels and need for dose adjustment. Additionally, a higher Vd than standard was previously reported in a historical cohort of adult patients with endocarditis [23]. We concluded that endocarditis might be a specific clinical entity for which development of a separate PPK model is warranted. Therefore, the aims of this study were: 1) to create a new PPK model for gentamicin treatment in endocarditis patients (further referred to as “the endocarditis model”); and 2) to assess whether the endocarditis model, and the models currently used in clinical practice, can accurately predict serum levels of gentamicin in endocarditis patients. Based on these results, one of the PPK models could be recommended for use.

## Materials and methods

MW\Pharm version 3.82 (Mediware, Groningen, the Netherlands) was used to perform PPK analyses, to develop the endocarditis model, and validate all three models in endocarditis patients.

### Patient data

A retrospective observational cohort study was performed with pharmacy data from patients treated at two Dutch university/tertiary hospitals: The Hague Hospitals and the University Medical Center Groningen (UMCG). Inclusion comprised patients ≥18 years of age, with data available about weight, height, gender, serum creatinine, gentamicin dosing and total gentamicin serum levels. Exclusion comprised patients with incomplete data, and patients on dialysis as this influences the gentamicin clearance. Medical records of all patients present in the hospital databases for gentamicin administration were studied to select eligible patients. If separate episodes of endocarditis were detected for a patient, these episodes were included as separate patients in our analyses. Data from The Hague Hospitals was used to develop a new PPK model (the modeling cohort; n = 65). Patients in the modeling cohort received intravenous gentamicin between 2011 and 2013. Data from the UMCG was used to validate the models (validation cohort; n = 14). Patients in the validation cohort received intravenous gentamicin between 2012 and 2014.

### Phase 1: Pharmacokinetic analysis and endocarditis model development

To develop the endocarditis model, the Kinpop module in MW\Pharm V3.82 was used to perform Iterative Two-Stage Bayesian (ITSB) analysis with serum levels from the modeling cohort. Inter-individual variability of the PK parameters was assumed to be distributed log-normally.

ITSB [25] needed initial estimates for each population parameter (mean and standard deviation (SD)) to start the iterative process. Thereafter, each patient’s PK parameters were determined based on its own measurements and the estimated population parameters as Bayesian priors. Then, the population mean and SD of each parameter were calculated from the patient’s parameters. These stages were repeated in the next cycle using previous population parameters as Bayesian priors, until the population parameters were fixed. ITSB has been shown to provide reasonable estimates for population parameters [19].

One-compartment models with first-order elimination were created, with estimates for metabolic/non-renal clearance (CLm), renal gentamicin clearance as a fraction of creatinine clearance (fr), and volume of distribution (Vd). For calculation of the clearance (CL) we chose the formula with total bodyweight (BW) and creatinine clearance of gentamicin according to Cockroft-Gault [26] (CLcr): CL = CLm*(BW/70) + fr*CLcr. This standard formula was chosen as it is clinically easy to use, serving applicability and simplicity for users. Furthermore, this approach enables a direct comparison as the ICU and standard models use this formula, too [24]. For volume of distribution, a unit of liter per kilogram of lean body mass according to the equation of Chennavasin [27] corrected for fat distribution [28] was used.

Different modified PPK models were designed, starting with population parameters provided by either the ICU or standard model. All newly designed PPK models were fitted to the data of the modeling cohort by testing different settings in MW\Pharm for the included PK parameters. Three settings exist in MW\Pharm for inclusion of pharmacokinetic parameters of a model: estimated with Bayesian prior (“Bayesian”), estimated with a predefined fixed population value (fixed population Bayesian, “FPB”), or set to a fixed value (“Fixed”).

The goodness-of-fit of the newly designed PPK models were evaluated using the Akaike Information criterion (AIC), and the weighted sum of squares of the residuals of concentration measurements and parameters (ΣWSS) divided by the degrees of freedom (df; the total number of measurements minus the number of estimated population and individual parameters) (ΣWSS/df). Both AIC and ΣWSS/df were aimed to be as close to zero as possible. The newly designed PPK model most reliably predicting gentamicin serum level in the modeling cohort (based on the AIC and ΣWSS/df closest to zero) was selected: the endocarditis model. To evaluate the significance of the covariates, a stepwise covariate analysis was performed, starting with a model without covariates. Nonparametric 95% confidence intervals of the population parameters for this model were obtained by bootstrap analysis with 1000 repetitions, which could be considered as a resampling technique for internal validation.

### Phase 2: Model validation and comparison

External validation of the standard, ICU, and endocarditis models was performed using patient data from the validation cohort, as this provides the strongest evidence for model validation. The Kinpop module in MW\Pharm was used with one cycle set as a maximum. In this setting, MW\Pharm determines the predictive power of a PPK model (a model’s ability to predict serum levels of an individual patient), as opposed to the iterative procedure for the fitting of a new PPK model to population data. CLm was fixed (“Fixed”) on a literature value [19,24], and for parameters fr and Vd Bayesian fitting was used (“FPB”).

The MDAPE (Median Absolute Prediction Error) and MDPE (Median Prediction Error) were used as criteria to assess which of the three models (the standard, ICU, or endocarditis model) predicted the serum levels of gentamicin most accurately in patients with endocarditis. In order to calculate the MDPE and MDAPE, measured gentamicin serum levels and predicted levels by the models were extracted from MW\Pharm. MDPE is a measure of bias, which is the median of the prediction errors for each serum sample. The predictive errors are calculated using: PE = (Cpredicted—C_observed_) / C_observed_. MDAPE is a measure of precision, which is the median of the absolute values of the prediction errors. Nonparametric 95% confidence intervals of MDPE and MDAPE were obtained by bootstrap analysis with 10,000 repetitions.

### Gentamicin assay

Gentamicin levels of serum samples drawn in the validation cohort before 2013, were determined using fluorescence polarization immunoassays on an AxSym automated analyzer (Abbott Laboratories, Chicago, IL, USA). Levels of serum samples drawn in the modeling cohort, and in the validation cohort after 2013, were determined using an enzyme multiplied immunoassay technique (EMIT) with an Architect Analyzer (Abbott Laboratories).

The lower limit of quantification for the EMIT technique was 0.2 mg/L. Since most samples were measured by the EMIT technique, the assay error for this technique was used for modeling. The assay error, describing the measuring error over the range of existent serum levels, is described by the following equation: SD = 0.0766 + 0.0006 C + 0.0064 C^2^.

### Ethical permission

A waiver was obtained for this research from the medical ethical committee in the UMCG for the act about Medical Research Involving Human Subjects (in Dutch: WMO) [date: July 29, 2014; file reference: M14.159588]. In addition, the board of directors of the Pharmacy The Hague Hospitals has approved the use of the anonymised data.

### Statistics

Demographic data of the modeling population and validation population were statistically compared in SPSS (IBM Predictive Analytics-software) using two sided Student’s t-tests. Equality of variances were tested with F-tests.

## Results

### Patient data

After patient selection, the modeling cohort contained 65 patients with 221 serum samples, and the validation cohort contained 14 patients with 30 serum samples. Only one patient from the validation cohort had two separate episodes of endocarditis. Table 1 shows the demographic data and clinical characteristics of the modeling and validation cohorts. There were no significant differences between the cohorts, with a limit for significance of p <0.05.

**Table 1:** Demographic data and clinical characteristics of the modeling and validation cohorts.

Characteristic	Modeling cohort (n = 65)		Validation cohort (n = 14)		
	Mean	Range	Mean	Range	P-value
**Age (years)**	69.3	32–92	63.4	30–88	0.12
**Weight (kg)**	76.2	46–121	80.3	65–90	0.12
**Height (cm)**	173.9	149–193	177.7	169–195	0.18
**Gender**	21F/44M	-	3F/11M	-	
**CLcr (ml/min)**	64.3	8.7–157.5	75.5	28.4–181.5	0.32
**CLcr (ml/min/1.73m**^**2**^**)**	58.4	7.8–153.2	65.6	23.9–141.1	0.47

CLcr = creatinine clearance, m^2^ = square meter body surface area. (S1 Data).

**Fig 1:**
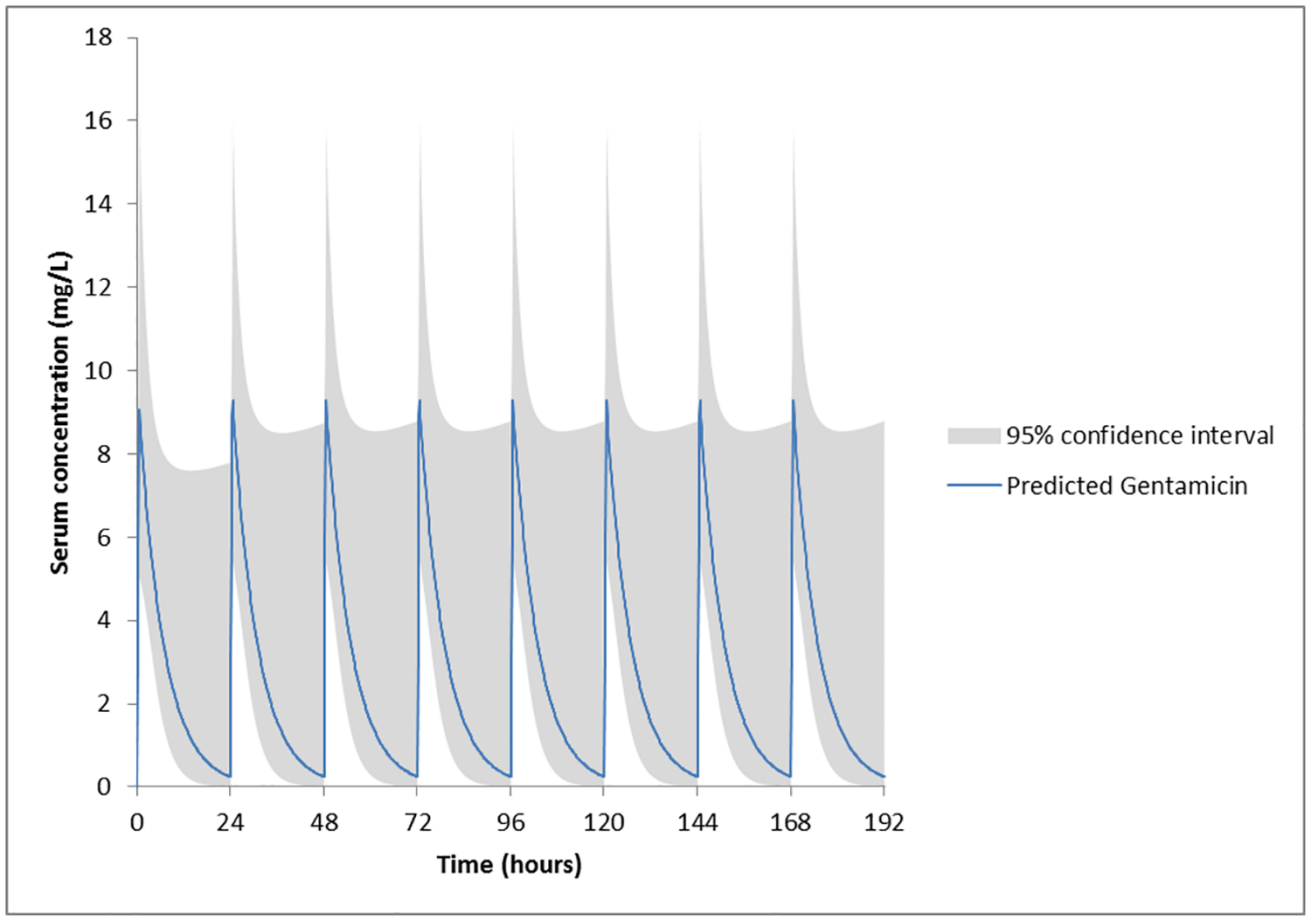
Simulation in the ICU model of a standard patient from the modeling cohort and the standard administration of 3 mg/kg gentamicin. Standard patient (see Table 1): male of 70 years old, 76.2 kg, and 174 cm, with a serum creatinin concentration of 86 μmol/L and a CLcr of 58 ml/min/1.73 m^2^. Fig 1 shows that the large variability in PK parameters results in a large variability in gentamicin serum levels between patients (see shaded area for 95% confidence interval). Therefore, TDM is needed from the first day of gentamicin administration.

**Fig 2:**
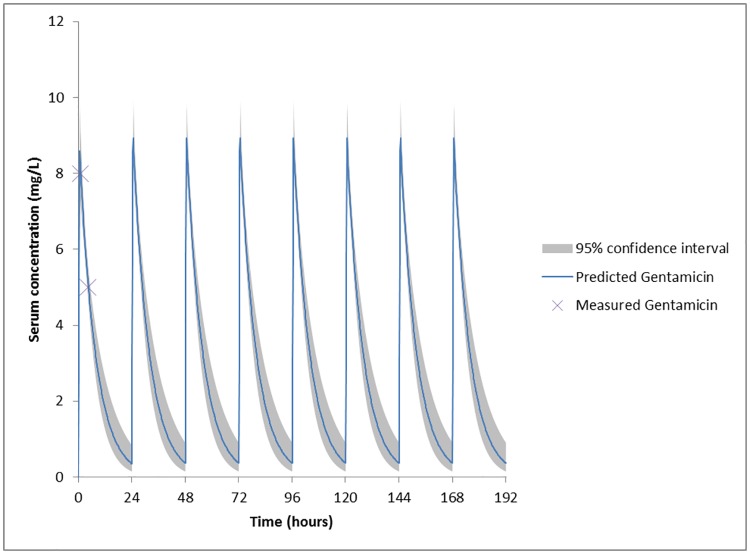
Bayesian simulation in the ICU model of a standard patient from the modeling cohort and the standard administration of 3 mg/kg gentamicin. Standard patient (see Table 1): male of 70 years old, 76.2 kg, and 174 cm, with a serum creatinin concentration of 86 μmol/L and a CLcr of 58 ml/min/1.73 m^2^. Prediction and spectacularly smaller 95% confidence interval (shaded area) as compared with Fig 1, for an individual based on two measurements of the gentamicin concentration in serum (purple cross): sample 1 on t = 1 h (30 minutes after the 30 minute infusion of gentamicin) with a concentration of 8 mg/L, and sample 2 on t = 4.5 h (4 hours after the 30 minutes infusion of gentamicin, with a concentration of 5 mg/L.

Fig 1 shows a simulation of the serum levels in the ICU model of a standard patient from the modeling cohort after the standard administration of 3 mg/kg gentamicin for endocarditis (blue line), with the 95% prediction interval (shaded area). It shows that the large variability in PK parameters results in a large variability in gentamicin serum levels between patients. Fig 2 shows the decrease in prediction uncertainty (shaded area) after TDM.

### Phase 1: Pharmacokinetic analysis and model development

Table 2 summarizes the ‘a priori’ population parameters used as starting point, the fitting settings, and the resulting AIC and ΣWSS/df values for all newly developed PPK models. The best fitting PPK model for the modeling cohort based on AIC and ΣWSS/df was parameterized with CLm fixed and Bayesian estimations for fr and Vd. The results of the stepwise covariate analysis (S1 Table), show that the model selected based on the description in the methods section results in the lowest AIC and thus should be regarded as the best model.

**Table 2:** Fitting settings used for model development with ITSB and the resulting parameters. In **bold** the selected endocarditis model, based on the AIC and ΣWSS/df as close to zero as possible. AIC = Akaike Information criterion, CLm = metabolic or non-renal clearance, fr = renal gentamicin clearance as a fraction of creatinine clearance, FPB = fixed population Bayesian, ICU = Intensive Care Unit, Vd = volume of distribution, ΣWSS/df = sum of the weighted sum of squares of the concentrations and parameters divided by the degrees of freedom. (S2 Data).

Population parameters	Settings			AIC	ΣWSS/df
	CLm	fr	Vd		
**ICU model**	Bayesian	Bayesian	Bayesian	1394	5.05
	FPB	Bayesian	Bayesian	1309	5.09
	**Fixed**	**Bayesian**	**Bayesian**	**1195**	**5.25**
**Standard model**	Bayesian	Bayesian	Bayesian	1394	5.05
	FPB	Bayesian	Bayesian	1306	5.08
	Fixed	Bayesian	Bayesian	1204	5.30

**Fig 3:**
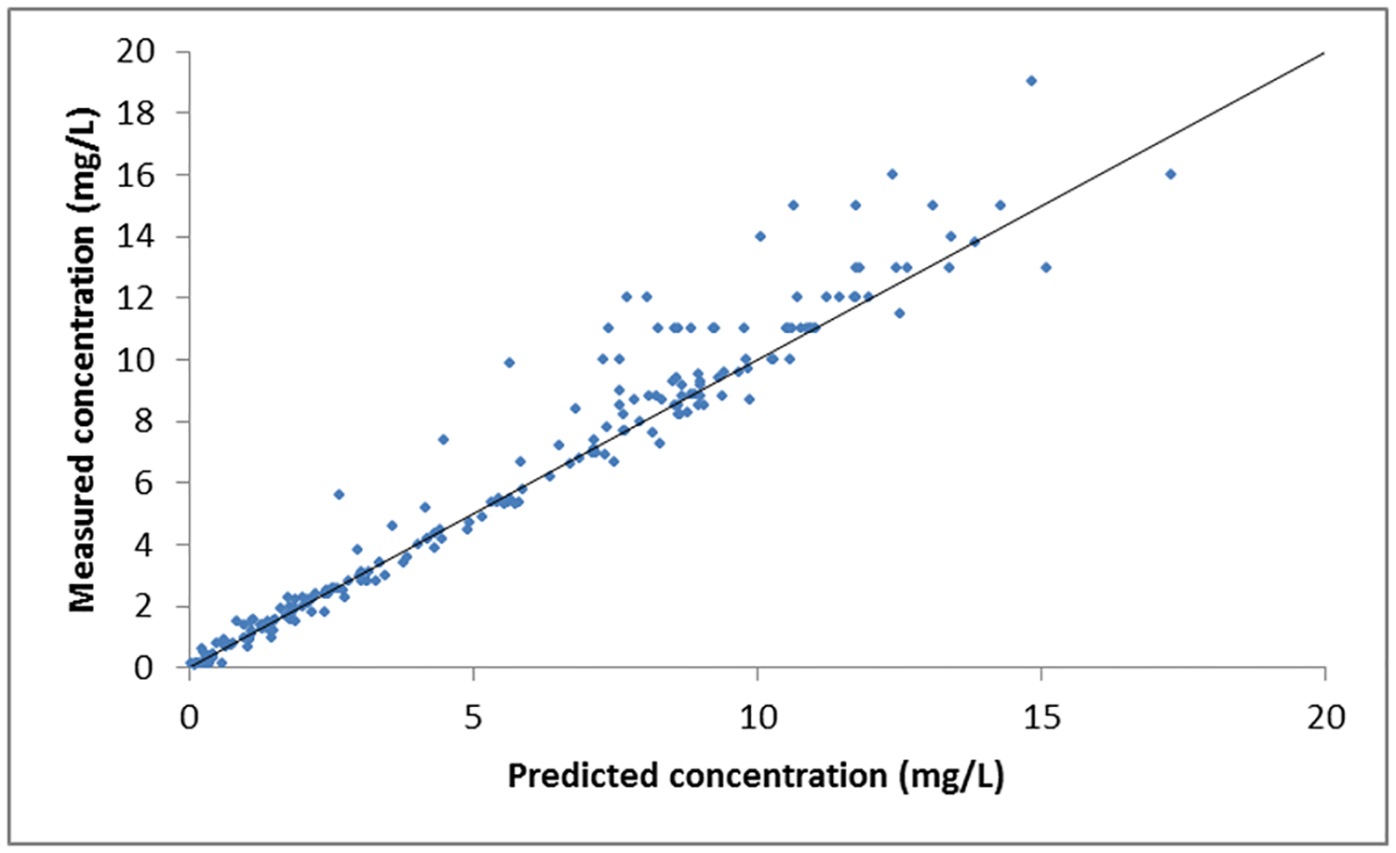
Diagnostic plot of measured concentrations versus predicted concentrations by the endocarditis model of gentamicin in the modeling cohort.

**Fig 4:**
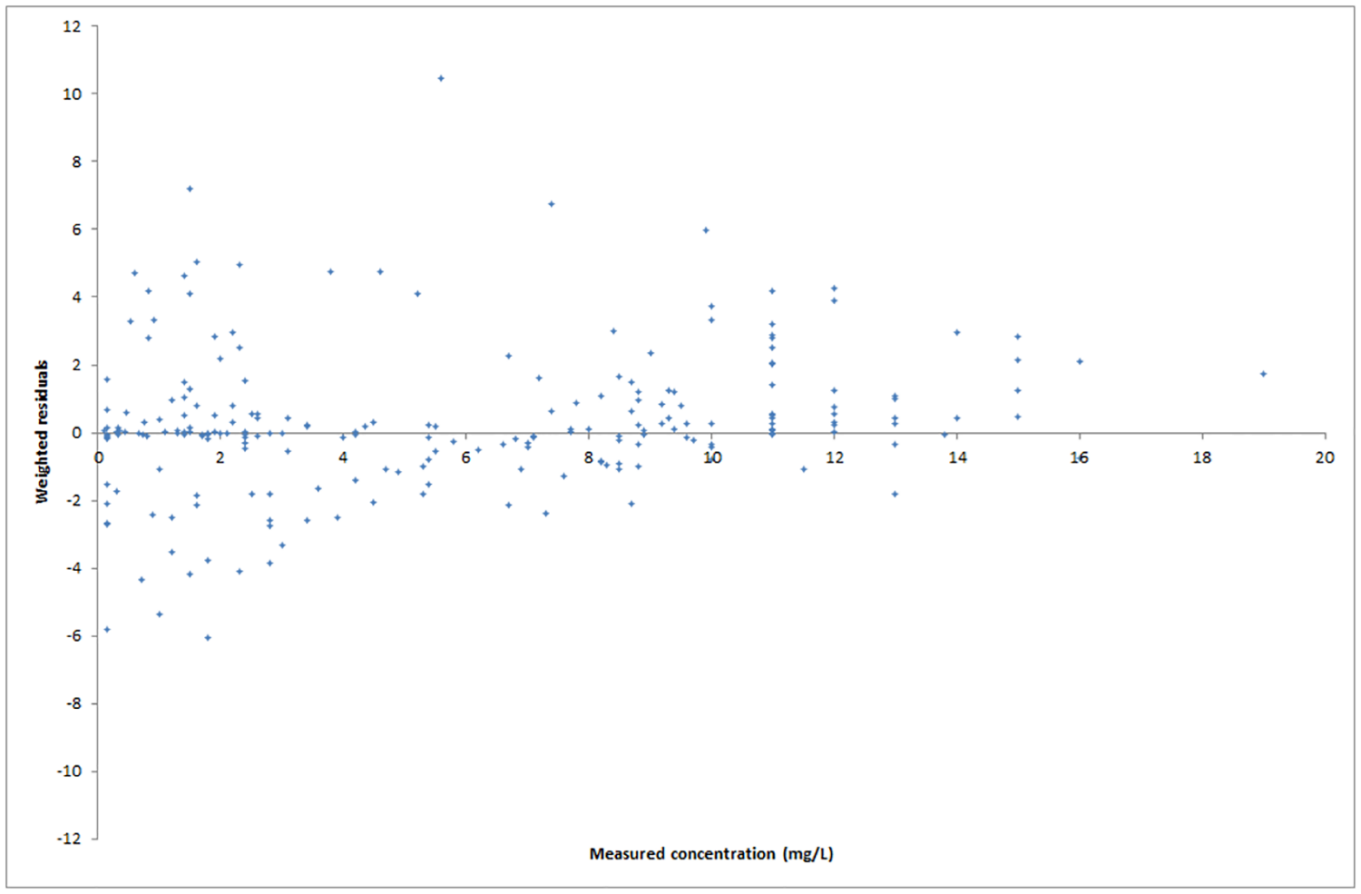
Diagnostic plot of weighted residuals (measured concentration minus predicted concentrations by the endocarditis model of gentamicin, divided by the standard deviation of the assay error) versus measured concentrations of gentamicin in the modeling cohort.

Diagnostic plots of measured concentrations versus predicted concentrations (Fig 3) and weighted residuals versus measured concentrations (Fig 4) showed a trend of the measured concentrations being higher than the predicted concentrations by the endocarditis model for concentrations above about 10–12 mg/L. There was no deflection of interest below this relevant range for infective endocarditis. This phenomenon was observed in the diagnostic plots of each of the models described in Table 2. Table 3 shows the estimated mean population parameters (± SD) for the selected endocarditis model, and for the clinically used ICU and standard models.

**Table 3:** Population parameters for gentamicin for ICU and standard models and the best newly developed endocarditis model (with nonparametric 95% confidence interval [CI] obtained by bootstrapping).

PK parameter	CLm (L/h/70kgBW)		fr		Vd (L/kgLBMc)	
	mean	SD	mean	SD	mean	SD
**ICU model**	0.277	0.138	0.899	0.417	0.335	0.104
**Standard model**	0.225	0.113	0.727	0.471	0.273	0.082
**Endocarditis model**	0.277	0	0.698	0.358	**0.312**	0.076
95% CI			[0.610; 0.794]	[0.273; 0.435]	[0.292; 0.331]	[0.060; 0.091]

BW = body weight, CLm = metabolic or non-renal clearance, fr = renal gentamicin clearance as a fraction of creatinine clearance, ICU = Intensive Care Unit, LBMc = lean body mass according to the equation of Chennavasin [27] corrected for fat distribution [28], SD = standard deviation, Vd = volume of distribution. (S2 Data).

### Phase 2: Model validation and comparison

**Fig 5:**
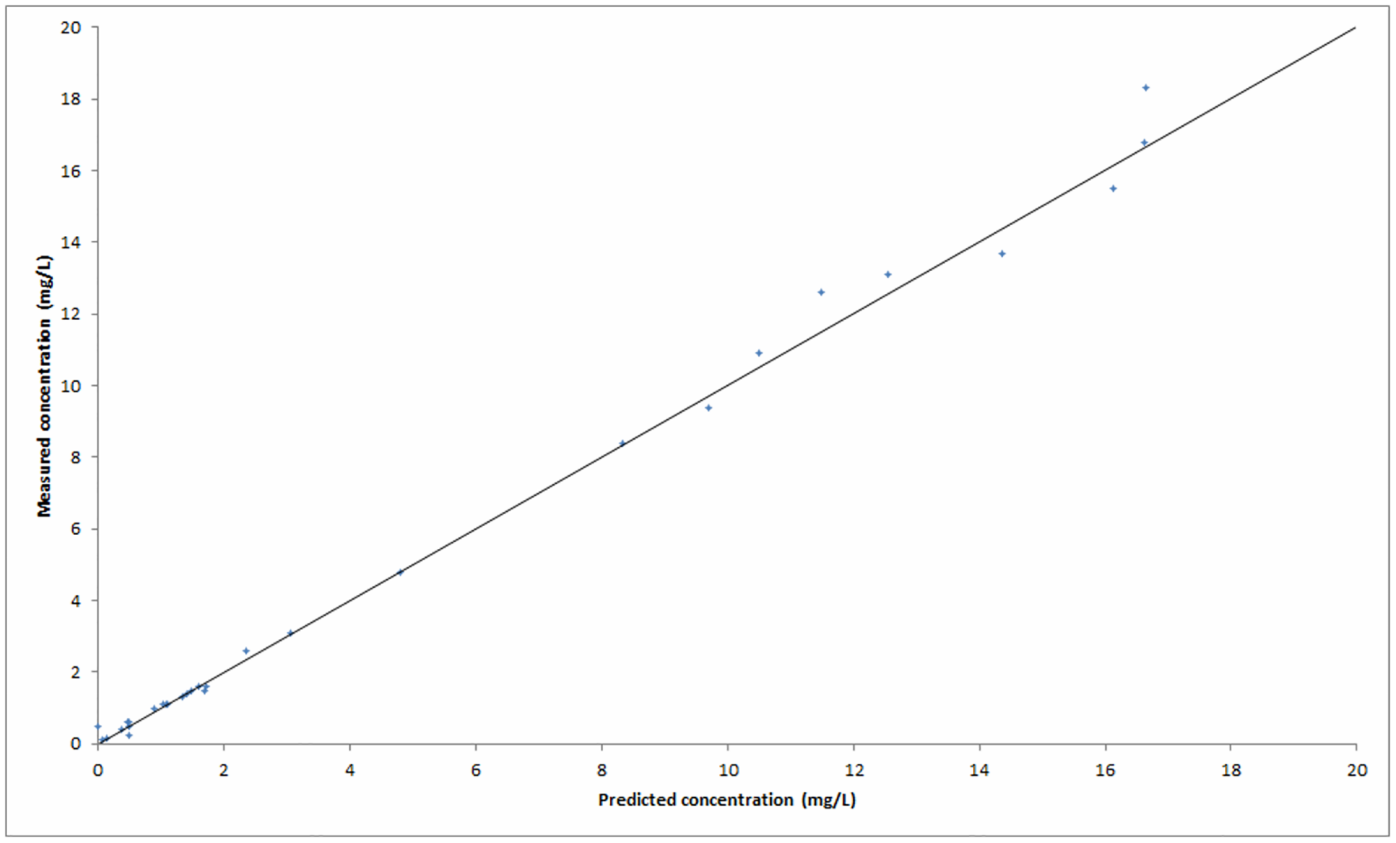
Diagnostic plot of measured concentrations versus predicted concentrations by the ICU model in the validation cohort.

The three PPK models (standard, ICU, endocarditis) were validated in the validation cohort. Table 4 shows the calculated MDPE and MDAPE. The ICU model yielded the best fit to the data of the validation cohort based on MDAPE. This held true for the standard model based on MDPE. As MDAPE is a reliable criterion, and to retain simplicity in clinical practice, the ICU model was selected as best model to accurately predict gentamicin serum level in endocarditis patients routinely. There was no trend of the measured concentrations visible in the validation cohort for either of the three models, as shown in Fig 5 for the ICU model.

**Table 4:** Validation parameters (with nonparametric 95% confidence interval [CI] obtained by bootstrapping) for comparison between models should be as close to zero as possible.

	ICU model	Standard model	Endocarditis model
**MDPE (bias)** 95% CI	-1.3% [-5.3; -0.5]	-0.9% [-4.5; 0.1]	-1.8% [-4.8; 0.1]
**MDAPE (precision)** 95% CI	4.4% [3.3; 9.0]	4.8% [1.8; 8.3]	4.7% [3.1; 9.0]

MDPE = Median Absolute Prediction Error, MDAPE = Absolute Median Prediction Error, ICU = Intensive Care Unit. (S3 Data).

## Discussion

The large inter individual variability seen in serum levels with a fixed starting dose of 3 mg/kg gentamicin (as depicted by the shaded area in Fig 1), and the spectacularly smaller variability seen with dosing based on two measured serum concentrations (as depicted by the shaded area in Fig 2), clarifies the need for TDM. TDM should start at day 1 of gentamicin administration, to increase the likelihood of therapeutic and non-toxic gentamicin levels as soon as possible. Therefore, a new PPK model for the prediction of gentamicin serum levels in endocarditis patients was created in this study using a modeling cohort. The endocarditis model was selected based on the observed goodness-of-fit (AIC and ΣWSS/df as close to zero as possible, see Table 2), presenting a CLm fixed at 0.277 l/h/70kgBW, and Bayesian fitting for fr at 0.698 (±0.358) and Vd at 0.312 L/kg LBMc (±0.076). During the external validation phase of this study, in which the three models were compared for their ability to predict serum gentamicin concentrations in the validation cohort, it was the ICU model showing the best fit (MDPE and MDAPE as close to zero as possible [29], see Table 4). Some points of this study are worth a discussion in more detail.

The parameterized Vd in the endocarditis model (0.312 ±0.076 L/kg LBMc) lies in between those of the standard (0.273 ±0.082 L/kg LBMc) and ICU (0.335 ±0.104 L/kg LBMc) models. Our present day cohort represents adult patients with endocarditis diagnosed according to the modified Duke criteria [30], with a relatively high percentage of intracardiac prosthetic material *in situ*, and treated with gentamicin dosed once daily. Similar findings were previously described by Rosell-Rovira *et al*. [23], who found a Vd of 0.29 L/kg in their historical cohort of adult patients with endocarditis according to the at the time conventional diagnostic criteria [31] and presumably treated with fractionated dosing of gentamicin. Even though our finding cannot be directly compared to the Vd reported by Rosell-Rovira *et al*. as measuring units differ, our data consolidate the evidence for a Vd of endocarditis patients being more similar to the Vd of ICU patients than to the Vd of standard ward patients, as they were previously reported for populations from the same geographical area, treated according to similar regimens and protocols, and investigated with comparable methodology [24]. This is important as the standard model is generally used for gentamicin dosing in endocarditis patients, and the presumed lower Vd in this model causes measured serum peak levels to be lower than predicted and required for therapeutic efficacy [19]. Potential complications of endocarditis underlying this increased Vd, include: 1) cardiac complications such as congestive heart failure [32], acute pericarditis and myocarditis [23]; and 2) infectious complications such as fever and sepsis [12].

The ITSB methodology used in our analysis is potentially less precise and newer methods, e.g. NONMEM, may have advantages as a methodology in purely scientific analyses but not in clinical practice. Offsetting, ITSB has been shown to compare well with several newer methods including NONMEM [25,33], and current clinically used TDM software is not able to work with NONMEM output directly. Therefore, to increase external validity and usability in clinical practice, we chose to use the ITSB method implemented in MW\Pharm as this output can be used directly in daily practice.

For a good estimation of CLm, patients with poor renal function have to be included in the modeling population. Inclusion of patients on dialysis would have provided such information. However, we chose to exclude these patients, as the extraction ratio of a dialysis membrane would add another significant uncertainty factor to the model. This extraction ratio needs estimation, depending on factors such as volume extraction, flow rate, the type and age of the dialysis membrane. Consequently, our modeling population contained only <10% of patients with a renal function <15ml/min and we were unable to estimate CLm reliably. Therefore, we chose to fix the parameter for CLm at a value previously determined in a comparable population [19,24], under the assumption that endocarditis would not affect metabolic clearance of gentamicin.

The performance of the endocarditis model in the modeling cohort differed from that in the validation cohort. This resulted from a large standard deviation (SD) for all estimated PK parameters in the endocarditis model and a relatively small validation cohort. Large SDs of PK parameters resulted from large inter individual variability in serum levels of gentamicin and omnipresent methodological errors which should be minimized by standardizing protocols (blood sampling, drug dose preparation, recording of the time point of gentamicin administration and blood sampling, gentamicin assay, data handling). Furthermore, as information about height and weight of patients is essential for accurate model development and validation, a considerable number of patients missing this information needed exclusion from our validation cohort.

Future research could cover the validation of the peak level range of 9–12 mg/l for once daily administration of gentamicin in endocarditis. This therapeutic target range is narrow [23], and has been extensively validated for fractionated dosing but not for once daily dosing. Of note, drug assays have been calibrated to yield the most reliable results within the level range normally found by fractionated dosing [16]. Future research could also focus on the (patho)physiology of the increased Vd in endocarditis patients, as we were unable to deduce this in our study.

Neither the standard, ICU, nor the endocarditis PPK model had clearly superior predictive power in our study. We conclude that the standard and ICU models are robust enough to resist variability of relevant characteristics in endocarditis patients, and at the same time provide acceptable predictive power for estimation of gentamicin serum levels. We advise to generally use the ICU model for gentamicin dosing in endocarditis patients, based on its best fit to the data of our validation cohort, the demand to treat endocarditis aggressively as soon as possible, and our ambition to maintain simplicity and thus safety in clinical practice. Taking into account the larger Vd found in endocarditis patients in the modeling phase of our study, and the fact that Vd prominently determines serum peak levels of gentamicin which in turn correlates with its bactericidal activity, endocarditis patients need higher gentamicin doses than advised by the standard model to prevent under dosing and to increase the likelihood of therapeutic efficacy.

## Supporting information

S1 Data (XLS)

S2 Data (XLS)

S3 Data (XLS)

S1 Table (DOCX)
