# Supplementary material for: Pharmacokinetic modeling of gentamicin in treatment of infective endocarditis: Model development and validation of existing models
Source: PLoS One. 2017 May 5;12(5):e0177324. doi: 10.1371/journal.pone.0177324 (PMC5419648; doi:10.1371/journal.pone.0177324)
Supplement: S1 Table — (DOCX) [file pone.0177324.s004.docx]

**S1 Table. Stepwise covariate analysis. In each step the covariate with the lowest AIC value (in bold) is included in the next step.**

| covariate | parameter | setting  CLm | setting  fr | setting  Vd | AIC | ΔAIC |
| --- | --- | --- | --- | --- | --- | --- |
| basic model |  | Bayesian | fr=0 fixed | Bayesian | 2060 |  |
| + BW | Vd | Bayesian | fr=0 fixed | Bayesian | 2036 | -24 |
| + LBM | Vd | Bayesian | fr=0 fixed | Bayesian | 2028 | -31 |
| + LBMc | Vd | Bayesian | fr=0 fixed | Bayesian | 2027 | -33 |
| + BW | CLm | Bayesian | fr=0 fixed | Bayesian | 2059 | -1 |
| + LBM | CLm | Bayesian | fr=0 fixed | Bayesian | 2051 | -9 |
| + LBMc | CLm | Bayesian | fr=0 fixed | Bayesian | 2054 | -6 |
| + CLcr | fr | CLm=0 fixed | Bayesian | Bayesian | 1285 | -775 |
| **+ CLcr** | **fr** | **fixed** | **Bayesian** | **Bayesian** | **1236** | **-823** |
| + BW | Vd | fixed | Bayesian | Bayesian | 1215 | -21 |
| + LBM | Vd | fixed | Bayesian | Bayesian | 1204 | -32 |
| **+ LBMc** | **Vd** | **fixed** | **Bayesian** | **Bayesian** | **1204** | **-32** |
| + BW | CLm | fixed | Bayesian | Bayesian | 1227 | -9 |
| + LBM | CLm | fixed | Bayesian | Bayesian | 1230 | -6 |
| + LBMc | CLm | fixed | Bayesian | Bayesian | 1229 | -8 |
| **+ BW** | **CLm** | **fixed** | **Bayesian** | **Bayesian** | **1195** | **-9** |
| + LBM | CLm | fixed | Bayesian | Bayesian | 1198 | -6 |
| + LBMc | CLm | fixed | Bayesian | Bayesian | 1197 | -8 |
